# Disinfectants

**Published:** 1885-02

**Authors:** 


### HALL'S JOURNAL OF HEALTH

VOL. 32. FEBRUARY, 1885. NO. 2.

### DISINFECTANTS.

In view of the fact that the cholera will probably visit the United States during the coming summer, everyone should prepare for its dreaded approach by a thorough and continued course of disinfecting.

This disease being one which is propagated by filth, and one which cannot ravage in a pure and unpolluted atmosphere, makes cleanliness a matter of life or death.

In setting out upon a course of cleansing and disinfecting a house, the first thing to be attended to is the drainage, for if this is defective, no amount of care will ward off the inevitable consequences.

Drains should be water and air tight throughout their entire length. A leaky or broken joint has killed entire families, by harboring the disease germs of typhoid and other fevers.

See that no sewer gas comes up through the waste-pipes and contaminates the air of sleeping-rooms.

An ounce of oil of pepermint poured into the waste-pipe will, if there be a leak, make its presence known by its odor at the point where it escapes from the drain.

Having made certain of perfect drainage, kalsomine or whitewash all walls that are not papered. Freshly slaked lime is a great purifier. On papered walls it would be well to spray, with an atomizer, some disinfecting fluid which will not stain, or the rooms might be closed up tightly and a quantity of sulphur burned in them. The stifling fumes make an excellent disinfectant. A dilute solution of carbolic acid may be used as a spray, but there seems to be considerable doubt about the efficacy of this agent for the purpose named.

A solution of corosive sublimate, of one-half grain to the ounce of water, is a powerful disinfectant, and also a deadly poison. If it is used at all it should be handled with caution. One-fourth of a grain of the pure substance might prove fatal if swallowed. Chlorine gas, which may be made by heating a mixture of sulphuric acid and black oxide of manganese, in a glass vessel over an alcohol flame, is much better than chloride of lime for fumigating a room, but a violent headache results from breathing the gas. All the foregoing methods should be used only where the room can be closed tightly during the process, and left for some time, to allow the fumes to do their work, before being opened; then a thorough airing should follow.

For throwing down waste-pipes and drains, to purify them, use a a strong solution of caustic potash. This dissolves the grease from the sides of the pipe, and should be followed by a solution of sulphate of iron, commonly known as copperas. This is a true disinfectant, is very cheap and has no odor. Most powders, sold under high-sounding names, are composed chiefly of this substance.

A very powerful disinfectant is permanganate of potash; it is, however, expensive, and leaves a dark stain on all cloth fabrics and on the skin

Above all have plenty of pure, fresh air. Open doors and windows, and let it blow into the house. After sleeping in a room, open the windows, remove the sheets and blankets from the bed, hang them on chairs, and wait an hour or two before having the bed made and closing the windows.

It is the neglect of little things and the disinclination to take a little extra trouble for the sake of cleanliness and health, that causes the majority of "ills that flesh is heir to."

